# Extensions to decision curve analysis, a novel method for evaluating diagnostic tests, prediction models and molecular markers

**DOI:** 10.1186/1472-6947-8-53

**Published:** 2008-11-26

**Authors:** Andrew J Vickers, Angel M Cronin, Elena B Elkin, Mithat Gonen

**Affiliations:** 1 Department of Epidemiology and Biostatistics, Memorial Sloan-Kettering Cancer Center, 307 East 63*rd *Street, New York, NY 10065, USA

## Abstract

**Background:**

Decision curve analysis is a novel method for evaluating diagnostic tests, prediction models and molecular markers. It combines the mathematical simplicity of accuracy measures, such as sensitivity and specificity, with the clinical applicability of decision analytic approaches. Most critically, decision curve analysis can be applied directly to a data set, and does not require the sort of external data on costs, benefits and preferences typically required by traditional decision analytic techniques.

**Methods:**

In this paper we present several extensions to decision curve analysis including correction for overfit, confidence intervals, application to censored data (including competing risk) and calculation of decision curves directly from predicted probabilities. All of these extensions are based on straightforward methods that have previously been described in the literature for application to analogous statistical techniques.

**Results:**

Simulation studies showed that repeated 10-fold crossvalidation provided the best method for correcting a decision curve for overfit. The method for applying decision curves to censored data had little bias and coverage was excellent; for competing risk, decision curves were appropriately affected by the incidence of the competing risk and the association between the competing risk and the predictor of interest. Calculation of decision curves directly from predicted probabilities led to a smoothing of the decision curve.

**Conclusion:**

Decision curve analysis can be easily extended to many of the applications common to performance measures for prediction models. Software to implement decision curve analysis is provided.

## Background

Clinical medicine has traditionally been divided into diagnosis, treatment and prognosis. From a research perspective, diagnosis and prognosis constitute a similar challenge: the clinician has some information and wants to know how this relates to the true patient state, whether this can be known currently (diagnosis) or only at some point in the future (prognosis). This information can take the form of a test – such as ultrasound for a blocked vein in the legs – or a statistical prediction model including several different variables. An example of the latter is the "Framingham risk calculator" which predicts death from cardiovascular disease on the basis of age, gender, smoking status, blood pressure and blood lipids[1]. Recent advances in medical biology, especially genomics, have raised the possibility that molecular markers might aid diagnosis or prediction. For example, it has been postulated that analysis of genes in breast cancer tissue can help predict whether breast cancer is likely to recur after surgery, and therefore whether chemotherapy would be of benefit[2].

### Decision analytic and biostatistical approaches to the evaluation of tests, models and markers

Decision curve analysis is a novel method for evaluating diagnostic tests, prediction models and molecular markers[3]. It was developed to overcome the limitations of traditional biostatistical methods on the one hand, and decision-analytic alternatives on the other. The traditional biostatistical approach to evaluating tests, models and markers focuses on accuracy, evaluating calibration and discrimination using metrics such as sensitivity, specificity or area-under-the-curve (AUC). Such methods are mathematically simple, can be used irrespective of whether the predictor is binary or continuous and generally have an intuitive interpretation. However, they have little clinical relevance. For example, how high an AUC is high enough to justify clinical use of a prediction model? Or take the case where a new diagnostic test increased specificity by 10% but decreased sensitivity by 5% compared to a standard test: should the new or old test be used?

Answering such questions depends on the consequences of the particular clinical decisions informed by the test, model or marker. In the case of the test that was more specific, but less sensitive, than the standard, its value depends on the harm of missing a case of disease relative to the harm of treating a patient unnecessarily. Decision-analytic methods can explicitly consider the clinical consequences of decisions. They therefore provide data about the clinical value of tests, models and markers, and can thus determine whether or not these should be used in patient care. Yet traditional decision-analytic methods have several important disadvantages that have limited their adoption in the clinical literature. First, the mathematical methods can be complex and difficult to explain to a clinical audience. Second, many predictors in medicine are continuous, such as a probability from a prognostic model or a serum level of a molecular marker, and such predictors can be difficult to incorporate into decision analysis. Third, and perhaps most critically, a comprehensive decision analysis usually requires information not found in the data set of a validation study, that is, the test outcomes, marker values or model predictions on a group of patients matched with their true outcome. In the principal example used in this paper, blood was taken immediately before a biopsy for prostate cancer and various molecular markers measured. The data set for the study consisted of the levels of the various markers and an indicator for whether the biopsy was positive or negative for cancer. A biostatistician could immediately analyze these data and provide an investigator with sensitivities, specificities and AUCs; a decision analyst would have to obtain additional data on the costs and harms of biopsy and the consequences of failing to undertake a biopsy in a patient with prostate cancer. Perhaps as a result, the number of papers that evaluate models and tests in terms of accuracy dwarfs those with a decision-analytic orientation.

### Decision curve analysis

Decision curve analysis has been described in prior methodologic[3] and conceptual papers[4]. In brief, the method is based on the principle that the relative harms of false positives (e.g. unnecessary biopsy) and false negatives (e.g. missed cancer) can be expressed in terms of a probability threshold. For example, if a man would opt for biopsy if he was told that his risk of prostate cancer was 20% or more, but not if his risk was less than 20%, it can be shown that he considers that harms associated with a missed cancer to be four times greater than the harms associated with an unnecessary biopsy, that is, the ratio of harms is the odds at the probability threshold[5]. This threshold probability can therefore be used to determine both whether a patient is defined as test-positive or negative and to model the clinical consequences of true and false positives using a clinical net benefit function:

$$\text{Net benefit}=\frac{\begin{array}{cc} True & Positives \end{array}}{n}-\frac{\begin{array}{cc} False & positives \end{array}}{n}\left(\frac{p_{t}}{1-p_{t}}\right)$$

where *n *is the total number of patients in the study and *p*_*t *_is the threshold probability. The threshold probability can then be varied to create the "decision curve" for any particular model, test or marker. The model, test or marker under study should first be converted to a predicted probability of the undesirable outcome (e.g. cancer on biopsy) denoted by $\hat{p}$: for a binary test, these probabilities are set to 1 and 0 for positive and negative test results; for a molecular marker, marker levels should be converted to a probability using logistic regression. The method of decision curve analysis is then as follows:

1. Select a *p*_*t*_

2. Define a patient as positive if $\hat{p}$ ≥ *p*_*t*_

3. Calculate the number of true and false positives

4. Calculate net benefit

5. Repeat for a range of *p*_*t*_

6. Repeat steps 1 – 5 for all models and for the strategy of treat all patients (i.e. $\hat{p}$ = 1)

**Figure 1:**
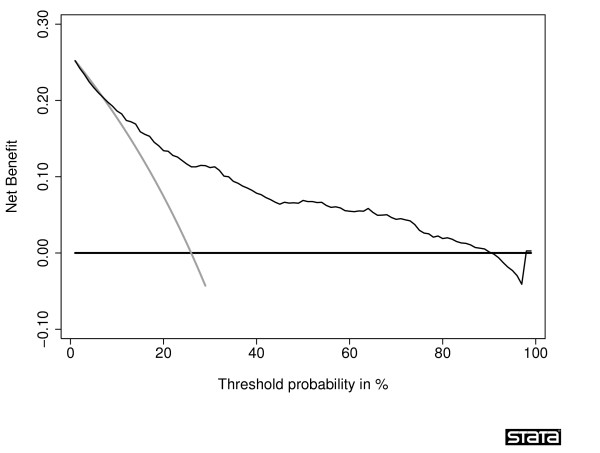
**Decision curve for a model predicting the outcome of prostate biopsy**. The thin grey line is the net benefit of biopsying all men; the thin black line is the net benefit of biopsying men on the basis of the statistical model; the thick black line is the net benefit of biopsying no man.

A typical decision curve is given in Figure 1. This curve was derived from a data set of men undergoing biopsy for prostate cancer and serves as the principal example for this paper. In brief, the data set included 740 men, never previously screened for prostate cancer, who were recommended for biopsy based on an elevated total PSA. Free PSA was also measured, and a digital rectal exam performed, on all men. Approximately one-quarter (n = 192) were diagnosed with cancer.

**Figure 2:**
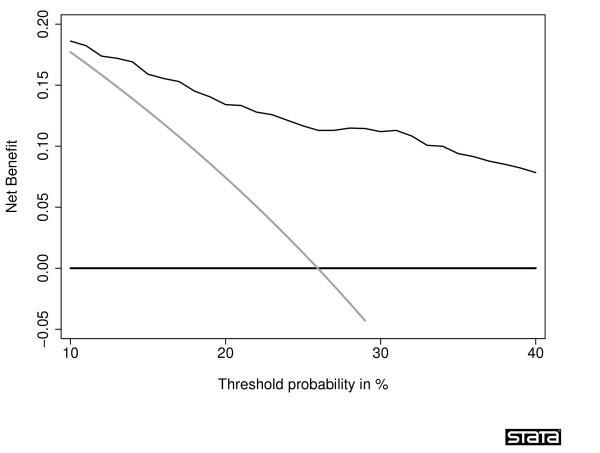
**Decision curve for a model predicting the outcome of prostate biopsy**. The thin grey line is the net benefit of biopsying all men; the thin black line is the net benefit of biopsying men on the basis of the statistical model; the thick black line is the net benefit of biopsying no man. The decision curve is shown for the key threshold probability range 10 – 40%.

Interpretation of the decision curve depends on comparing the net benefit of the test, model or marker with that of a strategy of "treat all" (the thin grey line) and "treat none" (parallel to the *x *axis at net benefit of zero). The strategy with the highest net benefit at a particular *p*_*t *_is optimal, irrespective of the size of the difference. Determining which men should be biopsied using the statistical model is superior to biopsying all men with elevated PSA once the threshold probability reaches about 10%, and is superior to the strategy of biopsying no man up to a threshold probability of about 90%. To interpret this result, one needs to consider the sort of probability for prostate cancer that men would need before they would decide to have a biopsy. A very risk averse man might opt for biopsy even if he had only a 10% risk of cancer. However, it seems unlikely that many men would demand, say, a 50% risk of cancer before they had a biopsy; this threshold would imply that an unnecessary biopsy is just as bad as a missed cancer. So one estimate for the range of *p*_*t*_'s in the community might be 10 – 40%. The net benefit of the decision curve for the statistical model is higher than that for either biopsying all or no men for all likely threshold probabilities (figure 2). This suggests that basing biopsy on the basis of our model will improve clinical outcome. Accordingly, decision curve analysis allows us to assess clinical relevance – which accuracy metrics cannot – without the need for additional data – as required by traditional decision-analytic approaches. The relative advantages and disadvantages of traditional biostatistical and decision-analytic approaches are described in Table 1, along with a comparison to decision curve analysis.

**Table 1:** Comparison of decision curve analysis with traditional statistical and decision-analytic methods

	**Traditional statistical analysis**	**Traditional decision analysis**	**Decision curve analysis**
**Mathematics**	Simple	Can be complex	Simple
**Additional data**	Not required	Patient preferences, treatment costs or effectiveness	General clinical estimates only
**Predictors**	Binary or continuous	Continuous predictors problematic	Binary or continuous
**Assess clinical value?**	No	Yes	Yes

We should note, however, that application of decision curve analysis comes relatively late in the development of a test, model or marker, once initial evaluations are complete and investigators are interested in understanding clinical consequences; indeed, a decision analytic evaluation of clinical value is often the last stage before clinical implementation. Biostatistical metrics are certainly key during earlier stages of development: for example, by assessing calibration and discrimination, those developing a statistical model can assess where improvements might need to be made.

### Limitations of decision curve analysis

Our initial paper on decision curve analysis was intended as an introduction to the method and did not include four critical aspects of model evaluation. First, models that are evaluated on the same data set that was used to build the model are at risk for overfit[6]. This can result in an overly optimistic evaluation of a model's value. As an example, take a 500 patient data set with an event rate of 10%, for which we develop a model with 20 variables, values of which are drawn randomly from a normal distribution. The model will typically have an AUC of around 0.70. Randomly selected numbers have no association with outcome and the AUC should be 0.50, reflecting poor discrimination of the model when applied to a data set of new patients.

One way to correct for overfit is to split the data into a "training" set, on which the model is constructed, and a "validation" set, on which the properties of the model, such as specificity, are calculated[7]. Although a robust and elegant solution to overfitting, splitting the data set reduces statistical power. A variety of statistical methods have been proposed that use the entire data set for both training and validation, but nonetheless correct for overfit. The two most common are cross-validation and bootstrapping[8]. In cross-validation, the data set is first randomly split into *K *groups. A model is then constructed using the data from the first *K*-1 groups and applied to the *K*^th ^group. The model building and validation process is repeated *K *times with each of the samples used once as the validation set. Accordingly, no patient is used both to develop and test a model. The idea of bootstrapping is to provide an estimate of the optimism associated with evaluating a model on the same data set that was used to develop it. This is achieved by creating training sets repeatedly by bootstrapping, building a model on the training set, and then calculating the difference in predictive accuracy between the model when applied to the training set and when applied to the validation set, which is simply the original data.

We have presented no method for correcting decision curve analysis for overfit. Hence it is entirely plausible that a method which has extremely poor discrimination after correction for overfit would appear to have clinical value in a decision curve analysis.

Second, in our initial paper on decision curve analysis, we presented no method for calculation of confidence intervals. It has been argued that confidence intervals have low relevance for decision-analytic methods. This is on the grounds that given a choice between two strategies, we should choose the one most likely to give us the best outcome, regardless of whether we believe it will be superior 51% or 99% of the time[9]. However, it is reasonable to suppose that, in some cases, clinicians will want to be sure that introduction of prediction model has a low chance of leading to inferior patient outcomes. This might be in the case where a well accepted clinical practice would be changed, for example, treating patients on the basis of a prediction model, rather than routinely treating all patients. Alternatively, a confidence interval might be used to inform the question of whether further research would be of value.

Third, we initially presented decision curve analysis only for binary outcomes. No method was provided for applying the method to censored ("survival time") data such as typically found in cancer studies.

Fourth, decision curve analysis requires a data set for which both patient outcome and the predictor for each patient are known. There may be situations where an analyst wishes to investigate a model on a data set where outcome is not known, such as the evaluation of a published statistical model on patients who have not been followed sufficiently. Similarly, for case control data, the values of the predictor are not known for all patients, only for cases and those selected as controls.

In this paper we present four extensions to decision curve analysis to address each of these issues: correction for overfit, calculation of confidence intervals, application to censored data and application directly to predicted probabilities.

### Correcting decision curves for model overfit

#### Methods

We tested the two most common methods of correcting for overfit, cross-validation and bootstrapping[10]. Techniques of cross-validation can vary with respect to the number of *K *folds; whether the cross-validation is conducted just once or *n *times with the results averaged over the *n *iterations; whether what is estimated for the *K*^*th *^group is the models' value (e.g. AUC), which is then averaged across *K *iterations, or a predicted probability for each patient, with the estimate for the model's value estimated just once using the predicted probabilities. We evaluated 10 and 2 fold cross-validation, on the grounds that these are the most common values for *K *and use repeated cross-validation. We also estimated probabilities rather than the decision curve for the *K*^*th *^group on computational grounds: a decision curve is a vector of net benefits at each threshold probability so we would need to save a vector for *K *groups and then average across groups.

We also investigated the value of bootstrap resampling correction for decision curve. For bootstrap correction we used the following steps:

1. Sample with replacement from the data set

2. Fit the model with the sample in (1)

3. Apply the fitted model in (2) to the sample in (1) to obtain the predicted probability of a prostate cancer diagnosis, and then compute the net benefit at various threshold probabilities.

4. Apply the fitted model (2) to the original data set to obtain the predicted probability of a prostate cancer diagnosis, and then compute the net benefit at various threshold probabilities.

5. Compute the difference in the net benefit obtained in (3) and (4) for each threshold probability.

6. Repeat steps (1) to (5) 200 times. Compute the mean difference in net benefit for each threshold probability across the 200 replications. This is the optimism.

7. The corrected net benefit for each threshold probability is the uncorrected net benefit minus the optimism from (6).

Repeated 10-fold cross-validation used the following steps:

1. Randomly divide the data set into 10 sets of equal size, ensuring equal numbers of events in each set

2. Fit the model leaving out the 1^st ^set

3. Apply the fitted model in (2) to the 1^st ^set to obtain the predicted probability of a prostate cancer diagnosis.

4. Repeat steps (2) to (3) leaving out and then applying the fitted model to the *i*th group, *i *= 2, 3... 10. Every subject now has a predicted probability of a prostate cancer diagnosis.

5. Using the predicted probabilities, compute the net benefit at various threshold probabilities.

6. Repeat steps (1) to (5) 200 times. The corrected net benefit for each threshold probability is the mean across the 200 replications.

Repeated 2-fold cross-validation is as for repeated 10-fold cross-validation, but with 2 sets instead of 10.

#### Data

Using the prostate biopsy data set described above, we used logistic regression to estimate the probability of a prostate cancer diagnosis with predictors of total PSA, free-to-total PSA ratio, age (>60 vs ≤ 60) and digital rectal exam result (abnormal vs normal). We dichotomized age so that the model would include two continuous and two categorical variables. We used the net benefit for this full sample as the gold standard (we describe this as the "best estimate" of net benefit). To artificially induce overfit, we randomly sampled from the data set such that we reduced the number of cancers to exactly *n*, where *n *took on values of 100, 50, 40, 30, and 20. In doing so, we kept the incidence the same (that is, when we sampled 100 cancers, there were 100/26% = 385 non-cancers). Using the predicted probability from the model, we estimated the net benefit at various threshold probabilities (15%, 25%, 35%, 60%, and 80%) with each data set. This gave us the uncorrected net benefit. We then calculated the corrected net benefits, using three methods: bootstrap, repeated 10-fold cross-validation, and repeated 2-fold cross-validation. The reported estimates of the uncorrected and corrected net benefits are the mean and 5^th ^to 95^th ^percentiles across 2000 replications.

## Results and discussion

The simulation results comparing the correction methods for the decision curve net benefits are shown in Table 2. For a threshold probability of 15%, the uncorrected estimate was over-optimistic for all scenarios; all correction methods gave an estimate lower than the best estimate of net benefit; repeated 10-fold cross-validation had the least bias for all but the scenario with 100 events, where the bootstrap estimate had slightly lower bias (-0.0001 vs -0.0005). Similar results were obtained for threshold probabilities of 25% and 35%. For the threshold probabilities of 60% and 80%, the bootstrap method had the least bias. The variability of the bootstrap and repeated 2-fold cross-validation methods was similar, however, the repeated 10-fold cross-validation method tended to have slightly less variability.

**Table 2:** Simulation results for correction for over-fit.

Threshold probability	Number of events (Total sample size)	"Best estimate" of net benefit	Uncorrected	Correction Method		
				
				Bootstrap	Repeated 2-fold	Repeated 10-fold
15%	100 (385)	0.1590	0.1618 (0.1438, 0.1799)	0.1589 (0.1406, 0.1771)	0.1569 (0.1412, 0.1734)	0.1585 (0.1425, 0.1755)
	50 (193)		0.1625 (0.1327, 0.1940)	0.1558 (0.1250, 0.1878)	0.1542 (0.1268, 0.1845)	0.1577 (0.1292, 0.1887)
	40 (154)		0.1625 (0.1288, 0.1972)	0.1540 (0.1181, 0.1902)	0.1517 (0.1185, 0.1877)	0.1565 (0.1222, 0.1937)
	30 (116)		0.1630 (0.1233, 0.2039)	0.1510 (0.1088, 0.1943)	0.1471 (0.1093, 0.1881)	0.1545 (0.1152, 0.1974)
	20 (77)		0.1670 (0.1155, 0.2224)	0.1484 (0.0946, 0.2044)	0.1341 (0.0877, 0.1837)	0.1509 (0.0998, 0.2061)

25%	100 (385)	0.1167	0.1216 (0.1021, 0.1420)	0.1172 (0.0977, 0.1381)	0.1160 (0.0988, 0.1342)	0.1174 (0.0990, 0.1363)
	50 (193)		0.1234 (0.0900, 0.1585)	0.1143 (0.0802, 0.1504)	0.1129 (0.0834, 0.1460)	0.1169 (0.0856, 0.1522)
	40 (154)		0.1243 (0.0861, 0.1640)	0.1131 (0.0733, 0.1541)	0.1104 (0.0747, 0.1483)	0.1155 (0.0787, 0.1556)
	30 (116)		0.1241 (0.0783, 0.1709)	0.1088 (0.0601, 0.1562)	0.1058 (0.0660, 0.1500)	0.1134 (0.0717, 0.1591)
	20 (77)		0.1297 (0.0741, 0.1892)	0.1069 (0.0474, 0.1679)	0.0918 (0.0411, 0.1440)	0.1098 (0.0529, 0.1712)

35%	100 (385)	0.0940	0.0967 (0.0744, 0.1189)	0.0916 (0.0692, 0.1139)	0.0893 (0.0715, 0.1087)	0.0916 (0.0720, 0.1121)
	50 (193)		0.0980 (0.0652, 0.1347)	0.0877 (0.0535, 0.1251)	0.0857 (0.0557, 0.1198)	0.0901 (0.0574, 0.1260)
	40 (154)		0.0993 (0.0602, 0.1411)	0.0865 (0.0455, 0.1297)	0.0829 (0.0456, 0.1212)	0.0883 (0.0495, 0.1281)
	30 (116)		0.0999 (0.0531, 0.1482)	0.0829 (0.0334, 0.1316)	0.0782 (0.0359, 0.1237)	0.0856 (0.0407, 0.1348)
	20 (77)		0.1048 (0.0472, 0.1665)	0.0795 (0.0178, 0.1421)	0.0636 (0.0134, 0.1162)	0.0822 (0.0238, 0.1436)

60%	100 (385)	0.0547	0.0568 (0.0377, 0.0765)	0.0498 (0.0302, 0.0699)	0.0446 (0.0275, 0.0628)	0.0495 (0.0320, 0.0688)
	50 (193)		0.0574 (0.0236, 0.0922)	0.0433 (0.0083, 0.0793)	0.0362 (0.0052, 0.0701)	0.0441 (0.0103, 0.0792)
	40 (154)		0.0593 (0.0181, 0.1007)	0.0421 (-0.0001, 0.0851)	0.0316 (-0.0045, 0.0708)	0.0405 (-0.0013, 0.0831)
	30 (116)		0.0608 (0.0123, 0.1116)	0.0384 (-0.0119, 0.0915)	0.0255 (-0.0152, 0.0715)	0.0373 (-0.0107, 0.0889)
	20 (77)		0.0650 (0.0060, 0.1286)	0.0324 (-0.0289, 0.0981)	0.0082 (-0.0392, 0.0610)	0.0291 (-0.0321, 0.0976)

80%	100 (385)	0.0189	0.0218 (0.0000, 0.0477)	0.0140 (-0.0068, 0.0391)	0.0104 (-0.0064, 0.0305)	0.0139 (-0.0032, 0.0350)
	50 (193)		0.0259 (-0.0148, 0.0670)	0.0100 (-0.0305, 0.0535)	0.0008 (-0.0305, 0.0377)	0.0100 (-0.0237, 0.0521)
	40 (154)		0.0287 (-0.0178, 0.0779)	0.0088 (-0.0381, 0.0581)	-0.0061 (-0.0424, 0.0357)	0.0068 (-0.0360, 0.0537)
	30 (116)		0.0351 (-0.0160, 0.0924)	0.0091 (-0.0436, 0.0638)	-0.0160 (-0.0611, 0.0354)	0.0022 (-0.0492, 0.0597)
	20 (77)		0.0404 (-0.0227, 0.1077)	0.0010 (-0.0672, 0.0714)	-0.0434 (-0.1057, 0.0199)	-0.0106 (-0.0872, 0.0678)

Values are mean (2.5^th ^– 97.5^th ^centile) of 2000 replications.

**Figure 3:**
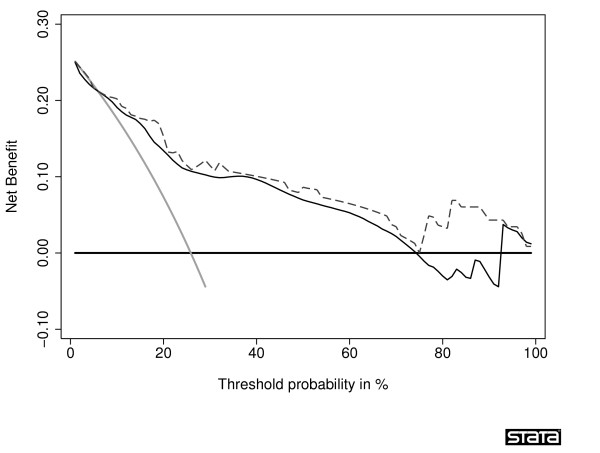
**Decision curve for a model predicting the outcome of prostate biopsy, with correction for overfit by crossvalidation**. The thin grey line is the net benefit of biopsying all men; the dashed black line is the net benefit of biopsying men on the basis of the statistical model; the thin black line is the results of the statistical model corrected for overfit; the thick black line is the net benefit of biopsying no man.

**Figure 4:**
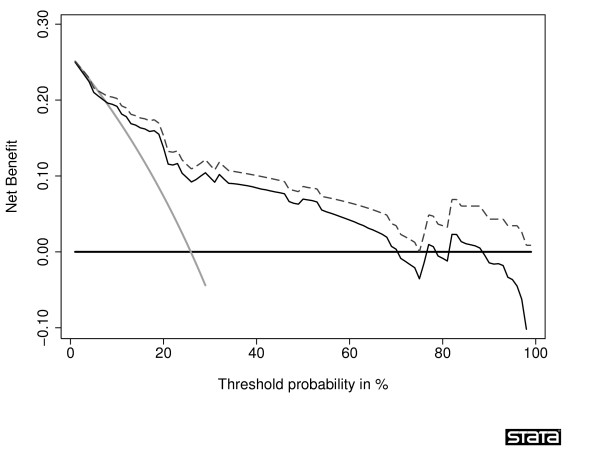
**Decision curve for a model predicting the outcome of prostate biopsy, with correction for overfit by bootstrap**. The thin grey line is the net benefit of biopsying all men; the dashed black line is the net benefit of biopsying men on the basis of the statistical model; the thin black line is the results of the statistical model corrected for overfit; the thick black line is the net benefit of biopsying no man.

A comparison of corrected net benefits from bootstrap and 10-fold cross-validation is shown in Table 3. In all comparisons for all threshold probabilities except 60% and 80%, the absolute difference in the corrected estimates was less than 0.005, with a relative difference in net benefit <6% (calculated as difference in net benefit divided by best estimate of net benefit). The 60% and 80% thresholds are near the tail of the decision curve, and are subject to excess random noise. The properties of the decision curve near this threshold are of minor interest because few men would require a ≥ 60% probability of cancer before they would accept biopsy. Thus the superior properties of the bootstrap at this threshold are of little value. To further examine the preferred correction method, we plotted sample decision curves with correction for overfit from a data set with 30 events (Figure 3 and Figure 4). One immediate attraction of repeated 10-fold cross-validation is that it has a smoothing effect on the decision curve. The curve remains unstable at very high threshold probabilities; however, these are rarely encountered in clinical practice (we rarely consider unnecessary treatment, say, 20 times worse than untreated disease). We therefore recommend repeated 10-fold cross-validation as a method to correct decision curves created using the same data as that used to generate the model.

**Table 3:** Simulation results for correction for over-fit: "best estimate" of net benefit minus net benefit after correction.

Threshold	Number of events	Correction method			Bootstrap – 10-fold
			
	(Total sample size)	Bootstrap	2-fold	10-fold	
15%	100 (385)	0.0001	0.0021	0.0005	-0.0004
	50 (193)	0.0032	0.0048	0.0013	0.0019
	40 (154)	0.0050	0.0073	0.0025	0.0025
	30 (116)	0.0080	0.0119	0.0045	0.0035
	20 (77)	0.0106	0.0249	0.0081	0.0025

25%	100 (385)	0.0005	0.0007	0.0007	-0.0002
	50 (193)	0.0024	0.0038	0.0002	0.0022
	40 (154)	0.0036	0.0063	0.0012	0.0024
	30 (116)	0.0079	0.0109	0.0033	0.0046
	20 (77)	0.0098	0.0249	0.0069	0.0029

35%	100 (385)	0.0024	0.0047	0.0024	0.0000
	50 (193)	0.0063	0.0083	0.0039	0.0024
	40 (154)	0.0075	0.0111	0.0057	0.0018
	30 (116)	0.0111	0.0158	0.0084	0.0027
	20 (77)	0.0145	0.0304	0.0118	0.0027

60%	100 (385)	0.0049	0.0101	0.0052	-0.0003
	50 (193)	0.0114	0.0185	0.0106	0.0008
	40 (154)	0.0126	0.0231	0.0142	-0.0016
	30 (116)	0.0163	0.0292	0.0174	-0.0011
	20 (77)	0.0223	0.0465	0.0256	-0.0033

80%	100 (385)	0.0049	0.0085	0.0050	-0.0001
	50 (193)	0.0089	0.0181	0.0089	0.0000
	40 (154)	0.0101	0.0250	0.0121	-0.0020
	30 (116)	0.0098	0.0349	0.0167	-0.0069
	20 (77)	0.0179	0.0623	0.0295	-0.0116

That said, we saw very little optimism where the number of events per variable was greater than 20, and thus do not see a strong justification for correcting decision curves for overfit where studies are of sufficient size. This will likely be the case for the sort of studies typically appropriate for decision curve analysis: we do not analyze small, preliminary studies to determine whether a test, marker or model would be of clinical benefit; evaluation of clinical effects is normally reserved for larger and more robust data sets.

### Confidence intervals for net benefits

A decision curve plot will have at least two curves and a straight line, and there will be many areas in which the curves overlap or are very close. Adding confidence bands to a plot, therefore, is likely to lead to confusing graph that is difficult to interpret. Accordingly, the best way to present confidence intervals for a decision curve analysis would be, first, to choose a limited number of key thresholds, and second, report the 95% C.I. for the difference in net benefit for pairwise comparisons between models at each of these thresholds.

#### Methods

We propose bootstrap methods, which are widely used and simple to implement, to obtain confidence intervals for the net benefit at a particular threshold.

1. Choose a limited number of threshold probabilities.

2. Sample with replacement from the data set

3. Fit the models of interest and compute the net benefits at threshold probabilities specified in (1) with the sample in (2)

4. Repeat steps (2) to (3) *n *times (we recommend *n *≥ 1000). The 95% confidence interval for the net benefit is given by the 2.5^th ^– 97.5^th ^percentiles across *n *replications.

It may be of interest to obtain the confidence interval for the difference in net benefit for two treatment strategies, for example, treating according to a model vs. treating all patients. In this case, in step 3 the difference in net benefit of those two treatment strategies should be computed.

#### Data

Logistic regression was used to estimate the predicted probability of a prostate cancer diagnosis. We fit one model with total PSA as the only predictor (the base model) and another model with total PSA, free-to-total PSA ratio, age and digital rectal exam result as the predictors (the full model). We used bootstrap methods to compute the confidence interval for three strategies: treat all patients, treat according to the base model, and treat according to the full model. We also computed the confidence interval around the difference in net benefit for the full model vs. treating all and full model vs. the base model.

#### Results

We obtained the confidence intervals for the net benefits associated with threshold probabilities of 15, 25, 35, 60, and 80% using bootstrap methods with 2000 replications (Table 4). Given are the point estimates of the net benefit for the three treatment strategies and the difference in full vs base and full vs all. The lower bound of the full model has a superior net benefit than both the base model and treating all for all threshold probabilities evaluated except 80%. We might therefore consider the value of the full model confirmed for the entire range of threshold probabilities that a man would typically require for a prostate biopsy.

**Table 4:** Confidence intervals for the net benefits using bootstrap methods.

Threshold	Net benefit	Point Estimate	Bootstrap Mean	Bootstrap confidence interval	
				
	For:		(2000 replications)	2.5^th ^percentile	97.5^th ^percentile
15	All	0.1288	0.1288	0.0922	0.1669
	Base	0.1288	0.1290	0.0922	0.1669
	Full	0.1590	0.1613	0.1280	0.1955
	Full vs All	0.0302	0.0325	0.0172	0.0490
	Full vs Base	0.0302	0.0322	0.0170	0.0488

25	All	0.0126	0.0126	-0.0288	0.0559
	Base	0.0748	0.0735	0.0432	0.1036
	Full	0.1167	0.1214	0.0919	0.1522
	Full vs All	0.1041	0.1088	0.0802	0.1369
	Full vs Base	0.0419	0.0479	0.0257	0.0712

35	All	-0.1393	-0.1393	-0.1871	-0.0894
	Base	0.0428	0.0410	0.0181	0.0662
	Full	0.0940	0.0965	0.0652	0.1291
	Full vs All	0.2333	0.2358	0.1990	0.2712
	Full vs Base	0.0511	0.0555	0.0293	0.0836

60	All	-0.8514	-0.8513	-0.9291	-0.7703
	Base	0.0149	0.0159	-0.0041	0.0378
	Full	0.0547	0.0569	0.0331	0.0838
	Full vs All	0.9061	0.9083	0.8345	0.9824
	Full vs Base	0.0399	0.0410	0.0176	0.0676

80	All	-2.7027	-2.7026	-2.8581	-2.5405
	Base	-0.0149	-0.0028	-0.0230	0.0243
	Full	0.0189	0.0223	-0.0054	0.0527
	Full vs All	2.7216	2.7249	2.5649	2.8757
	Full vs Base	0.0338	0.0251	0.0000	0.0595

### Application of decision curve analysis to censored data

Calculation of net benefit for a decision curve requires an estimate of the rate of true and false positives. For survival time data, this requires that survival time must be converted to a binary endpoint at a prespecified landmark time, for example, patient alive at five years. However, survival data are typically subject to censoring: a man who entered a study, say, three years before the analysis was conducted and was alive at that time is "censored" because we know he lived more than three years, but not how much longer.

One solution is to exclude patients who were event free at last follow-up but whose survival time is less than our landmark time. This is associated with two problems. The first is that informative data are removed from analysis: a patient who was censored at 4 years and 11 months most likely survived to 5 years but is treated identically in the analysis as a patient followed for only one month. Second, removing censored patients from the analysis increases the prevalence of the event. This is because patients followed for less than 5 years will be counted if they die but not if they survive. Changing the prevalence is important because it affects the proportion of true and false positives, and therefore the net benefit.

#### Methods

To calculate the net benefit for survival time data subject to censoring, we first define x = 1 if the patient has a predicted probability from the model ≥ *p*_*t *_(the threshold probability) and x = 0 otherwise; s(t) is the Kaplan-Meier survival probability at our chosen landmark time t, and *n *is the number of subjects in the data set. Using methods similar to Begg et al[11], the number of true positives is given by [1 - (s(t) | x = 1)] × P(x = 1) × *n *and the false positives as (s(t) | x = 1) × P(x = 1) × *n*. Naturally, one assumption of the method is that the mechanism of censoring is independent from the predictors used to create the model.

Heagerty et al[12] point out that this method can, in some instances, result in a non-monotone relationship between the predicted probability from the model and sensitivity or specificity. Yet there is no requirement that a decision curve be monotone by *p*_*t*_: there is no inherent contradiction in having net benefit increase above some *p*_*t *_= *k*, and then decrease at some *p*_*t *_= *l *for *l *> *k*. Indeed, this is often what is seen in the right-hand tail of the decision curve, where there is a relatively limited number of cases, and the curve is subject to excess sampling variation. Nonetheless, the rationale for decision curve analysis is to evaluate the clinical effects of a test, model or marker. Studies aiming to affect clinical practice tend to be large, and should be well populated across the threshold probabilities of interest. As such, we should expect the important parts of the decision curve to be monotone.

In time-to-event analyses where the failure event is something other than death, it is often important to consider the effects of competing risks. A competing risk is any event that a subject could experience, that would alter the likelihood of having the event of interest. The most common competing risk is death before the event of interest, such as recurrence of cancer, since a subject cannot experience the event of interest after they die. In the presence of competing risks, the cumulative incidence function, which takes into account the probability of having the competing risk event, can be used to estimate the probability of having the event of interest [13]. To calculate the net benefit in the presence of competing risks, we denote the cumulative incidence of the event of interest by time t as *I*(t). The number of true positives is given by (*I*(t) | x = 1) × P(x = 1) × *n *and the false positives as [1 - (*I*(t) | x = 1)] × P(x = 1) × *n*. That is, we use the same formula as in the absence of competing risks, but using the estimate from the cumulative incidence function in place of the Kaplan-Meier estimate. It is known that the probability of the event calculated using Kaplan-Meier methods is generally higher than when taking into account competing risks [14]. We therefore expect that, in general, net benefit will be lower when competing risks are taken into account.

#### Simulation study without competing risks

We conducted a simulation study with 2000 replications to check the method for computing the net benefit for survival time data in the absence of competing risks. We simulated data with 5000 subjects and created a binary predictor *x *(1 if positive and 0 if negative) and generated an event time *T*_*i *_for each subject *i *such that *T*_*i *_was related to *x*. We then generated a uniform censoring time *C*_*i *_for each subject *i *and defined the observed time for subject *i *as the minimum of *T*_*i *_and *C*_*i*_, denoted by *Y*_*i*_. We determined the coverage of the method described above, for a given time *t *and for a threshold probabilities of 15, 30, and 60%. Coverage was defined as the proportion of 95% confidence intervals, calculated using bootstrap methods described above, that contained the true net benefit. To obtain the true net benefit, we simulated data in the same way but with an arbitrarily large data set and *Y*_*i *_equal to *T*_*i*_. Due to the absence of censoring, the true positives are subjects with *x *= 1 and *T*_*i *_<*t *and the false positives are subjects with *x *= 1 and *T*_*i *_> *t*. We conducted simulations for three time-points *t *and with three relationships between the predictor and event: the predictor equally sensitive and specific, the predictor more specific, and the predictor more sensitive. Approximately 10%, 20%, and 30% of patients were censored, respectively, before time-point 1, 2, and 3.

#### Results of simulation study without competing risk

Results of the simulation study where the predictor was equally sensitive and specific are given in Table 5. For all scenarios, there was little bias and coverage was excellent. For example, for a threshold probability of 15%, a predictor being equally sensitivity and specific to the event, and evaluated at timepoint 1, the true net benefit was 0.0185 and the mean net benefit over 2000 replications was 0.0186. Similar results were obtained for the simulations where the predictor was more specific and where the predictor was more sensitive (data not shown).

**Table 5:** Simulation results for a survival-time endpoint.

Threshold	Timepoint	True Net benefit	Mean estimate	Coverage (%)
15%	1	0.0185	0.0186	95.0
	2	0.1171	0.1169	94.7
	3	0.2177	0.2172	95.0

30%	1	-0.0526	-0.0524	95.3
	2	0.0672	0.0670	94.5
	3	0.1894	0.1888	95.6

60%	1	-0.3546	-0.3543	94.5
	2	-0.1450	-0.1453	94.5
	3	0.0688	0.0678	95.3

**Figure 5:**
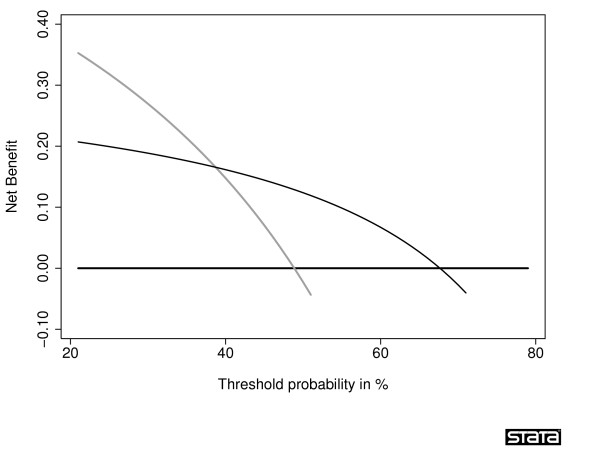
**Decision curves for survival time data**. The thick grey line is the net benefit for a strategy of treating all men; the thick black line is the net benefit of treating no men. A thin grey line is calculated from an uncensored data set for a binary variable of survival at time *t; *a thin black line is calculated from the data set after censoring was introduced, using the net benefit formula for censored data. The two curves are essentially overlapping and appear as a single dark grey line.

A decision curve from a survival time data set with 30% censoring is shown in figure 5. To create this figure, we used an uncensored survival time data set, created a binary outcome for survival at *t*, and calculated net benefit for binary data. We then applied censoring as described above, and calculated a second decision curve calculating net benefit for censored data. The two curves are essentially overlapping, suggesting good properties of our method.

#### Data for censored data with competing risks

We used two previously studied data sets to demonstrate the effects of competing risks on decision curve analysis. The first data set contained 4462 bladder cancer patients who underwent radical cystectomy [15]. The event of interest was recurrence (1068 events). Since bladder cancer patients tend to have significant comorbid conditions, 846 patients died from other causes without recurrence, which was considered the competing risk event. We calculated the decision curve for a multivariable prediction model (the "bladder nomogram") with and without adjustment for competing risks[15]. Age is one of the predictors in the model and is strongly associated with the death from other causes. We therefore expected the decision curves with and without adjustment for competing risks to be different.

The second data set contained 7765 prostate cancer patients treated by radical prostatectomy [16]. Similar to the bladder cancer data set, the event of interest was recurrence and the competing risk event was death from other causes without recurrence. Prostate cancer patients tend to be in otherwise good health, only 368 patients died without recurrence, while 1256 patients recurred. We calculated the decision curve for a multivariable model including PSA, stage, and grade. As the competing risk was rare, and the predictors for recurrence unassociated with the competing risk of death, we expected the decision curves with and without adjustment for competing risk to be very similar.

#### Results for survival time data with competing risks

**Figure 6:**
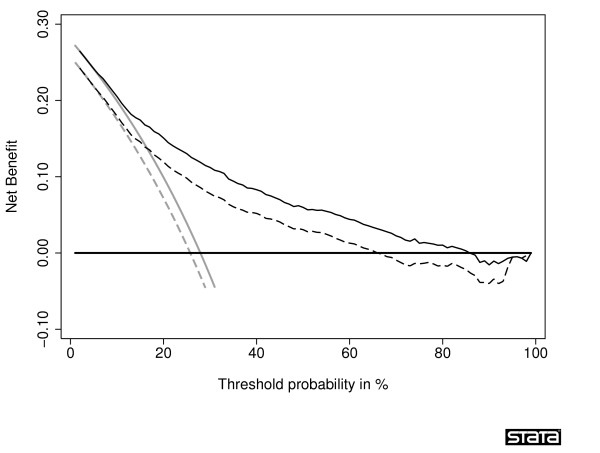
**Decision curve for survival time data with and without adjustment for competing risk, where the incidence of competing risks is high (bladder cancer data set)**. The thick grey line is the net benefit for a strategy of treating all patients with (dashed line) and without (solid line) adjustment for competing risk; the thin black line is the net benefit of a strategy of treating patients according to the model with (dashed line) and without (solid line) adjustment for competing risk; the thick black line is the net benefit of treating no patients.

**Figure 7:**
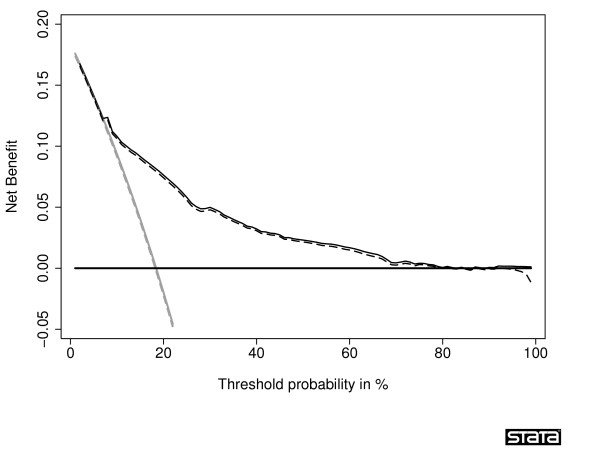
**Decision curve for survival time data with and without adjustment for competing risk, where the incidence of competing risks is low (prostate cancer data set)**. The thick grey line is the net benefit for a strategy of treating all men with (dashed line) and without (solid line) adjustment for competing risk; the thin black line is the net benefit of a strategy of treating men according to the model with (dashed line) and without (solid line) adjustment for competing risk; the thick black line is the net benefit of treating no men. Since the incidence of competing risk is low, the curves for treating all are essentially overlapping and appear as a single grey line.

Decision curves with and without adjustment for competing risk are shown in figure 6 and figure 7. In the bladder cancer example – where the incidence of the competing risk is high, and the predictor is associated with the competing risk – we do see, as expected, that adjustment for competing risk lowers net benefit for both the model and for the strategy of "treat all". However, decisions about the value of the model are not likely to be affected because the model remains of value over a wide range of threshold probabilities. In the prostate cancer example – where the incidence of the competing risk is low, and the predictor unassociated with the competing risk – the decision curves with and without adjustment for competing risk are essentially overlapping.

### Application of decision curve analysis when outcome or predictor data are not available

We may sometimes want to calculate decision curves in the absence of outcome data. For example, a statistical model is published in the literature and is shown to be well-calibrated. An investigator wishes to know whether application of the model would be of clinical benefit, either because this was not reported by the original authors, or because the investigator believes that the properties of the model may differ for the population that he or she is interested in, because the distribution of predictors may vary between populations. The model predicts some future event, such as cancer incidence or recurrence, and the investigator's data set is relatively immature, with few patients followed for a sufficient period of time.

Alternatively, we may wish to calculate a decision curve in the absence of predictors. This would occur in a case-control study, where predictors are only measured on a proportion of patients without the event.

If a model is well calibrated, that is, if close to *x*% of a sample of patients with a predicted risk of *x*% have the event, true and false positives can be calculated directly from predicted probabilities. Using ${\hat{p}}_{i}$ as the predicted probability for the *i*^th ^patient, where *m *> 0 patients have $\hat{p}$ ≥ *p*_*t*_, net benefit is calculated as:

$$\text{Net benefit}=\sum_{i=1}^{m}{\hat{p}}_{i}-\sum_{i=1}^{m}\left(1-{\hat{p}}_{i}\right)\left(\frac{p_{t}}{1-p_{t}}\right)$$

**Figure 8:**
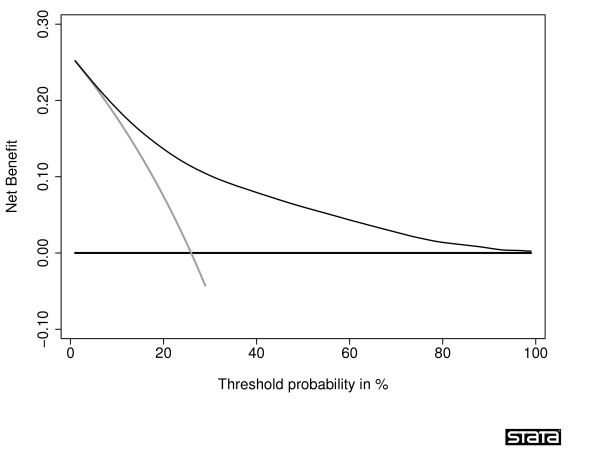
Decision curve for complete data set calculated directly from predicted probabilities.

A decision curve for the principal example, calculated using this formulation rather than outcome data, is given in figure 8. The curve is not subject to sampling noise and so has a smooth shape.

### Statistical code for decision curve analysis

We have written statistical code to implement decision curve analysis and its extensions. In Stata, we have created two commands: *dca *for a binary outcome and *stdca *for a survival-time outcome; corresponding Stata ado and help files are available for both commands. For *dca*, the user inputs a binary outcome variable and one or more predictor variables. Within the command, the user has the option to plot the decision curve or save the points of the decision curve to a Stata data file. To calculate a decision curve in the absence of outcome data, the user specifies the predicted probability from the model as both the outcome and the predictor variable. For *stdca*, the user inputs the predictor variables (the data must already be declared as survival-time data using *stset*) and a timepoint of interest. The output is similar to that of *dca*. In R, we have created two R functions: dca.R and stdca.R. These functions are implemented similar to the Stata commands, however, in stdca.R the user must also specify as inputs the time and failure variables. The Stata and R code can be found at  along with tutorials on using the code (including survival time data, multivariable models, joint and conditional models), discussions of how to interpret decision curves, and code to implement correction for overfit by repeated 10-fold cross-validation.

## Conclusion

In this paper, we have described four extensions to decision curve analysis: correction for overfit, confidence intervals, application to time-to-event data and application to data sets where outcome or predictor data are unknown. All of these extensions are based on straightforward methods that have previously been described in the literature for application to analogous statistical techniques.

## Competing interests

The authors declare that they have no competing interests.

## Authors' contributions

AV conceived of the paper and guided the statistical studies; AC ran and advised on the statistical methods; EE advised on decision-analytic aspects of the paper; MG advised on statistical methods.

## Pre-publication history

The pre-publication history for this paper can be accessed here:


